# Supplementary material for: Prediction of Intracranial Infection in Patients under External Ventricular Drainage and Neurological Intensive Care: A Multicenter Retrospective Cohort Study
Source: J Clin Med. 2022 Jul 8;11(14):3973. doi: 10.3390/jcm11143973 (PMC9317602; doi:10.3390/jcm11143973)
Supplement: Supplementary file 1 [file jcm-11-03973-s001.zip › jcm-1756485-supplementary.pdf]

Supplementary Table S1. Demographic and clinical features between training-set and validation-set*

Variable	Training set (n=483)	validation set (n=111)	*p*
Clinical characteristics			
Gender (male)	216 (44.72%)	51 (45.95%)	0.815
Age (years)	54.6(14.91)	56.6 (13.02)	0.193
BMI	23.44 (3.72)	23.29 (3.59)	0.704
History of diabetes	70 (14.49%)	17 (15.32%)	0.825
ASA grades	2.44 (0.92)	2.51 (0.96)	0.49
BP on admission (mmHg)	141.91 (12.47)	141.56 (12.62)	0.795
GCS 3-8	72 (14.91)%	18 (16.22%)	0.73
GCS 9-12	85 (17.60%)	22 (19.82%)	0.583
GCS 13-15	326 (67.49%)	71 (63.96%)	0.476
Pre-operative intubation (n, %)	97 (20.08%)	19 (17.12%)	0.48
Lopt (minutes)	129.89 (81.64)	143.36 (85.01)	0.121
Cases underwent operations in addition to EVD (n, %)	186 (38.51%)	51 (45.95%)	0.149
Diagnosis and complications			
Hydrocephalus (n, %)	57 (11.8%)	16 (14.41%)	0.45
Spontaneous ICH (n, %)	259 (53.62%)	57 (51.35%)	0.665
Traumatic brain injury (n, %)	162 (33.54%)	43 (38.74%)	0.299
Skull fracture (n, %)	145 (30.02%)	32 (28.83%)	0.804
tSAH (n, %)	177 (36.6%)	38 (34.2%)	0.634
CSF leakage due to trauma (n, %)	28 (5.8%)	8 (7.21%)	0.574
Non-intracranial infections (n, %)	31 (6.42%)	9 (8.11%)	0.522
First laboratory tests			
RBC (10^{12}/L)	4.49 (0.84)	4.51 (0.86)	0.877
HB (g/L)	119.02 (31.05)	115.73 (44.04)	0.179
WBC (10^{9}/L)	13.16 (5.02)	12.71(4.59)	0.391
NEUT (%)	84.53 (9.54)	83.79 (10.32)	0.477
PLT (10^{9}/L)	199.86 (70.38)	195.08 (67.35)	0.516
TBIL (μmol/L)	12.4 (6.93)	12.65 (6.97)	0.737
DBIL(μml/L)	4.92 (2.89)	5.02 (2.88)	0.748
ALT (U/L)	34.144 (27.003)	38.259 (29.086)	0.161
AST (U/L)	37.911 (31.491)	39.917 (37.121)	0.565
LDH (U/L)	204.92 (64.79)	201.89 (59.24)	0.653
HDL (mmol/L)	2.03 (0.59)	2.03 (0.58)	0.895
LDL (mmol/L)	2.93 (1.17)	2.97 (1.12)	0.539
Ch (μml/L)	5.02 (1.14)	4.91 (1.07)	0.331
Ab (g/L)	37.177 (7.353)	36.110 (7.115)	0.17
GLB (g/L)	33.3 (9.78)	29.9 (7.68)	0.775
BUN (mmol/L)	5.92 (1.72)	5.95 (1.75)	0.839
UA (mmol/L)	248.64 (94.89)	264.98 (98.23)	0.105
SCR (μmol/L)	5.99 (1.18)	5.99 (1.07)	0.686
Post-operative EVD monitoring			

Length of EVD (days)	6.07 (2.24)	5.72 (2.34)	0.142
Number of CSF sampling (per week)	2.99 (1.37)	2.99 (1.39)	0.97
Leakage from EVD site (n, %)	32 (6.63%)	11 (9.91%)	0.229
Outcomes			
ICU length of stay (days)	8.2 (4.53)	7.69 (3.78)	0.274
Hospital stays (days)	15.04 (6.78)	14.41 (6.02)	0.634
In-hospital mortality (n, %)	61 (12.63%)	12 (10.81%)	0.599

*Continuous data are shown as mean (standard deviation).

Abbreviations: EVD, external ventricular drainage; CSF, cerebropinal fluid; ICH, intracranial hemorrhage; ASA, American Society of Anesthesiologists; Lopt, length of operation time; tSAH, traumatic subarachnoid hemorrhage; RBC, red blood cell; HB, hemoglobin; WBC, white blood cell; NEUT, neutrophil ration; PLT, platelet; TBIL, indirect bilirubin; DBIL, direct bilirubin; ALT, glutamic pyruvic transaminase; AST, glutamic oxalacetic transaminase; LDH, lactate dehydrogenase; HDL, high-density lipoprotein; LDL, low-density lipoprotein; Ch, cholinesterase; Ab, albumin; GLB, globulin; BUN, urea nitrogen; UA, uric acid; SCR, creatinine; BP, blood pressure; GCS, Glasgow coma scale; BMI, body mass index; ICU, intensive care unit.

Supplementary Table S2. Characteristic of patients with tSAH

**Variable**		tSAH (n=215)	Non-tSAH (n=379)	**P-value**
	3-8	49 (22.79%)	22 (5.80%)	＜0.01
**GCS**	9-12	87 (40.47%)	28 (7.39%)	
	13-15	162 (75.35%)	226 (59.63%)	
**Hospital stays (days)**		15.67 (3.43)	9.78 (3.54)	＜0.01
**Length of EVD (days)**		18.85 (6.14)	12.05 (3.24)	＜0.01
**Time to infection (days)**		7.94 (1.32)	5.99 (0.82)	＜0.01
**ICU length of stay (days)**		7.49 (1.42)	5.07 (1.44)	＜0.01

Abbreviations: tSAH, traumatic subarachnoid hemorrhage; GCS, Glasgow Coma Scale; EVD, external ventricular drain; ICU, intensive care unit.
